# Abstract of the Proceedings of the Buffalo Medical Association

**Published:** 1864-06

**Authors:** Joseph A. Peters

**Affiliations:** Secretary


BUFFALO

### Medical and Surgical Journal.

VOL. III. JUNE, 1864. No. 11.

ART. I.—*Abstract of the Proceedings of the Buffalo Medical Association.*

TUESDAY EVENING, May 3d, 1864.

Society met pursuant to adjournment, the newly elected President, Dr. Samo, in the Chair. Present, Drs. White, Rochester, Miner, Strong, Congar, Wetmore, Ring, Johnson and Peters.

On taking the chair the President begged to be excused from making a speech, but in a few words returned his thanks for the honor done him, and expressed his determination to discharge the duties of his office to the best of his ability.

On motion of Dr. White the reading of the minutes was dispensed with.

Moved that Drs. H. Van Guysling, O. H. C. Burger and E. B. Tefft, be invited to take part in the proceedings of the Association until such time as they shall have completed their memberships in the Erie County Medical Society. Carried.

Voluntary communications being in order,

*Dr. White* wished to exhibit to the members a vial of specimens which he considered to be cholesterine or cholesterine and bile. Had given some of them to Prof. Hadley for examination, but had not yet received his report, still he had no doubt as to their character, they answered so fully the descriptions of *biliary calculi.* Their history, which he would give, rendered them very interesting to him. About three years ago the patient from whom they were taken, a lady twenty-six years old, was delivered of twins—her first confinement—and was soon afterwards attacked with puerperal mania, and was brought to this city for his treatment. He found on examination uterine enlargement, with incomplete involution of the organ. The *os tincae* was ulcerated, and the lining membrane inflamed. Her intelligence seemed entirely gone, there being a total neglect of all her duties, and loss of all knowledge of her position. Gave her tonics, and treated the local disease topically, and her mind was soon restored simultaneously with the cure of the uterine disease. From that time he saw her but two or three times until August of last year, when there existed a tumor of the abdomen about half way belween the umbilicus and the *mons veneris*, and about an inch to the right of the *linea alba*. This tumor was about the size of a large orange, and was somewhat nearer the umbilicus than the *mons*, seemed attached to the abdominal parietes and was very painful. Had supposed it to be connected with the uterus, and would confess himself very much surprised when, after two or three months it ruptured and discharged a semi-purulent and albuminoid fluid, together with the specimens shown, which he regarded as biliary calculi. The size of the tumor has diminished, the general health of the patient is improved, her insanity has not returned, and she attends to all her household duties, though a fistulous opening remains in the hypogastric region through which the semi-purulent fluid and these concretions are from time to time discharged.—The gall bladder must have been in some way tapped, but he was utterly unable to say how. The liver was not enlarged; there was no icterode, nor were there any symptoms to call attention to the liver, until these calculi were discharged. Had not ventured to explore it much; had once or twice passed a whalebone probe a short distance into the opening, and had dilated the opening occasionally to assist the passage of the calculi.—Regarded it as a very remarkable case, and had seen no mention of a similar one.

*Dr. Congar* had supposed cholesterine was not confined to the liver, but might be found in nearly every organ. Its production is universally ascribed to the liver, but he would like to inquire whether it was not carried in the blood to the most remote parts of the body? Dr. Austin Flint jr. in a recent article speaks of a kind of blood-poisoning from this source, it having been found after death in several different organs of the body. Any source of irritation in these cases might become an outlet for the cholesterine just as it does sometimes for tubercle or other blood poison.

*Dr. Rochester* said that he thought a precisely similar case was mentioned by Stromeyer, where cholesterine was found in a tumor of the abdomen of a woman—as also in the testicle of a man—upon examination however, he finds Stromeyer does not state that the cholesterine was found in the form of biliary calculi. *Crystals* of cholesterine are certainly found in nearly all tumors, had seen some himself a few days since in examining a fatty tumor. Biliary matter may certainly be absorbed into the blood, as we all know occurs when an obstruction takes place in the duodenum, the coloring matter of the bile being absorbed, and giving rise to the icterode appearance of the conjunctivæ and skin. Biliary matter with cholesterine is mentioned as occurring in the urinary bladder. Was inclined to think these specimens were biliary calculi and came from the gall bladder.

*Dr. White* knew that cholesterine was found in other parts of the body than the liver, but did not know that biliary calculi were. These concretions when cut open present a laminated appearance with nuclei, the laminæ being white and yellow. Thinks there is no doubt that these are biliary calculi. Might mention, not to seem excessively ignorant, that he knew that the absorption of cholesterine is mentioned by Symond in his work on Organic Chemistry.

*Dr. Wetmore* mentioned having taken from the gall bladder of a subject on the dissecting table 420 biliary calculi, of a good size, which were counted, besides many smaller ones not counted. They were precisely similar in appearance to the specimens shown by Dr. White.

*Dr. Miner* had found crystals of cholesterine in a fatty tumor—one species of tumor had been named by Cruveilheir from this circumstance. Has seen a case where the gall bladder was completely filled, containing from an ounce and a half to two ounces of perfectly black calculi. The woman in whose gall bladder they were found had become as black as a negro, though originally white. Her eyes however were jaundiced, which was not the case with the negro, and the skin very much roughened. She died with ascites from the obstruction of the portal circulation, and on post mortem examination he could find no disease of the liver or any other organ except as stated. Disease of the liver may have existed, and he failed to detect it from lack of experience in observation at that time. It would appear probable that such condition would be associated with manifest disease of that organ.

*Dr. White* discharged a patient on Saturday last who had been brought here from Oswego for his attendance. She had been confined about six months ago, and was informed by her attendant, a Homœpath, that she took cold, and had inflammation of the womb. About two months ago she was brought here a raving maniac, it requiring two relatives, strong men, to restrain her. Found the uterus enlarged, inflamed, and emitting a copious yellow discharge. The long diameter of the uterus by measuremen was almost four inches, but when discharged it was less than two and three-fourths inches. She was placed under the care of the Sisters of Charity, and treated with tonics, mild laxatives, open air, and local applications. An issue was also burned in the anterior neck. In addition to her other troubles she also had an hysterical paralysis of the right arm and leg, and muscles of mastication and deglutition. She now is able to walk, reads and writes, and is perfectly conscious, though her mind is yet feeble. The local ulcerations are nearly healed, and menstruation has taken place, though whether it had before, or not, could not be ascertained. He had mentioned this case for the purpose of calling attention to the connection between the uterine disease and the deranged condition of the mind. Many more such cases might be enumerated, but this would suffice. Believed a large proportion of cases of mania in females to be of the kind enumerated, and thought the cause too generally overlooked, and its investigation too much neglected. Had found in all such cases that mental improvement took place *pari passu* with improvement locally. Would mention the following prescription, which he had used with good results in these cases:

℞ Pot. Bromid. - - - - - ℥ ss.

Ferri Pyrophos. - - - - - ʒi.

Aquæ, - - - - - - f ℥ viij.

Fiat solutio.

Dose—teaspoonful three times daily.

*Dr. Rochester* while on the subject of uterine diseases would mention a case of some interest which had occurred in his practice. A lady, 40 years of age, who had borne three children, one of whom is living, and who supposed herself to be about three months pregnant, went to spend the night at the bedside of a sick friend. About 3 o'clock in the morning she was attacked with a very sudden and severe pain in the abdomen, together with a slight uterine hemorrhage. Believing herself about to miscarry she hastened home and he was sent for, but being out of town a Homœpathic practitioner was called in. Twenty-four hours afterwards, having returned, he (Dr. R.) saw her and found the Homœpath had left her, saying she was dying. Found her pulseless, cold, almost insensible, but suffering the most agonizing pain in the abdomen, and vomiting almost incessantly. She could not retain the slightest thing on her stomach. Tried stimulants, morphia, champagne, etc., but without avail. It was impossible to move her enough to administer enemata, so it was finally concluded to try hypodermic injection, which was accordingly done, and one-fourth of a grain of morphia was thrown under the skin of the abdomen. The effect was almost instantaneous, in less than ten minutes she felt sleepy, in half an hour she took some champagne, and from that time the case progressed steadily to recovery. He would give his hypothesis in regard to it, which was that during ovarian excitement, (of course the patient was mistaken in supposing herself pregnant,) hemorrhage occurred into the cavity of the abdomen, causing peritonitis. The uterus was inflamed, with a strawberry color of the orifice. He found it difficult to pass the sound on account of some obstruction, the passage being followed by a few drops of dark colored blood. The after treatment consisted of quinine and morphia in *full* doses. Supposed this to be the same kind of case as sometimes resulted in the formation of pelvic hematocele. He considered it very interesting in its use, progress, and termination, as well as affording an illustration of the value of hypodermic injection.

*Dr. White* called attention several years ago to the danger of making injections into the womb, and had long since abandoned the practice. The most harmless fluids often produce a train of symptoms resembling those related by Dr. Rochester, (or what had been called "womb colic,") owing as he supposed to the passage of a portion of the fluid into the cavity of the abdomen. This result would even follow the injection of warm water, while the crayon of pure caustic would not lead to any such result.—Wished to compliment Dr. Rochester on his treatment in the case reported, and on the ingenuity of the hypothesis by which he accounted for it.

*Dr. Strong* was bound to doubt whether blood or any other fluid could exist in any quantity within the cavity of the abdomen, and the patient recover so readily, and without graver symptoms being produced than Dr. Rochester had narrated.

*Dr. Rochester* wished to correct any misapprehension which might exist in the minds of gentlemen present, in regard to the case he had reported, by saying that it was a very severe case indeed, and the symptoms as grave, and the recovery as difficult as could well be imagined.

*Dr. Strong* had no wish to criticise the diagnosis, or treatment of the case related, but was bound to doubt the hypothesis by which it was attempted to explain it. Thought the symptoms might follow from less grave causes.

Some further discussion here took place, upon this point, which was participated in by Drs. White, Rochester and Strong.

*Dr. Rochester* would relate the case of a patient, a young gentleman, who had shown him occasionally for the past year or two a tumor on the ring finger of his left hand. The patient ascribed it to an injury received from working on the brakes of a fire engine, and sometime since painted it with tincture of iodine, which destroyed the skin and showed underneath a languid growth which pressed out the little finger. In that condition he came to Dr. Rochester, who made a free incision, as he supposed, to the bone, not meeting with any obstruction he measured the distance on the outside and finding he had passed the point where the bone ought to have been, decided it to be enchondroma, a cartilaginous growth from the medullary centre of bones, and requiring amputation, from its liability to recur. Accordingly a few days ago, assisted by Dr. Boardman, he administered chloroform, and made a longitudinal incision upon the mass, and found a cartilaginous mass, connected on the upper side of the phalanx with a ring of bone. The tumor could have been removed, but as that would give no security against its recurrence, it was decided to amputate, which was done through the lower phalanx. On examination it was found to consist of a spongy growth from the medullary portion of the bone, and underneath the flexor tendons, on the lower surface was another similar and independent tumor, about the size of a split pea. Had seen on two occasions at Bellevue removals of enchondromata of eight years duration. Believed that surgeons were agreed that it was necessary to remove the bone in such cases.

Reports on prevailing diseases being in order, Dr. Strong reported having seen a good deal of pharyngitis and laryngitis. Dr. Rochester had noticed some diarrhœa of a dysenteric character.

Some discussion took place as to the relative prevalence of diptheria in the country towns and in this city, it seeming to be a fact that it had been more frequent in the former, than the latter locality.

After the transaction of considerable miscellaneous business, it was moved and carried that Dr. John Trowbridge be allowed to resume his connection with the Society on paying fees, and Dr. Ayer, who was proposed at the March meeting, was elected a member on complying with the by-laws.

Adjourned.

JOSEPH A. PETERS, *Secretary*.

